# 713. Coccidioidal Meningitis Among Children: A Case Series and Single Center Experience In Central California

**DOI:** 10.1093/ofid/ofab466.910

**Published:** 2021-12-04

**Authors:** Fouzia Naeem, Linda Giglio, Julia Sharma, Patricia Clerkin, Frederick Laningham, James McCarty

**Affiliations:** 1 Valley Children's Healthcare, Madera, California; 2 Dartmouth-Hitchcock Medical Center, Lebanon, New Hampshire; 3 Stanford University School of Medicine, Stanford, California

## Abstract

**Background:**

Coccidioidal meningitis is a severe form of coccidioidomycosis associated with significant morbidity and mortality. Published literature in the pediatric population is limited, particularly on coccidioidal meningitis. Here we describe a large case series of pediatric coccidioidal meningitis followed at a tertiary care center in an endemic region.

**Methods:**

We performed a retrospective case review of patients ≤21 years old followed at our facility with a diagnosis of coccidioidal meningitis from January 1, 2000, to December 31, 2018.

**Results:**

Overall, 30 patients were identified during the study period. The median age was 10.8 years (IQR: 4.6-15). The majority of patients were previously healthy (93%) and all required hospitalization. Fever (90%), headache (70%), vomiting (53%), and fatigue (57%) were the most common clinical manifestations. More than one-third (40%) had concurrent pulmonary disease. Only 20 patients (67%) had initial *Coccidioides* complement fixation (CF) titers >=1:16. The majority had extra-axial brain involvement (60%) and seven (23%) had associated spinal canal disease. Over two-third required shunt placement (70%) and almost half of them (43%) underwent revision. Neurological complications including paresis/paralysis, stroke, neuropathy, seizures, and cognitive delay were observed in 20% of patients. Two-thirds (73%) of patients received fluconazole as the initial drug. However, 37% of those had fluconazole failure, requiring alternative treatment. Due to refractory disease, two patients required a novel triazole, isavuconazole, while adjunctive therapy with steroids and interferon-gamma (IFNγ) was used in 20% of patients. Most cases (83%) stabilized, 13% experienced relapses and/or progressive disease, and 3% were fatal.

**Conclusion:**

Pediatric coccidioidal meningitis is an uncommon and sometimes devastating complication of disseminated coccidioidomycosis. Many patients present with relatively low CF titers, and communicating hydrocephalus and long term neurologic complications are common. Fluconazole treatment failures are common, and management remains difficult despite recent advances in therapy. Most patients do well once the disease is stabilized and require lifelong therapy. Newer therapeutic agents are needed.

**Disclosures:**

**All Authors**: No reported disclosures

